# Evaluation of movements of lower limbs in non-professional ballet dancers: hip abduction and flexion

**DOI:** 10.1186/1758-2555-3-16

**Published:** 2011-08-05

**Authors:** Erica E Valenti, Vitor E Valenti, Celso Ferreira, Luiz Carlos M Vanderlei, Oseas F Moura Filho, Tatiana Dias de Carvalho, Nadir Tassi, Marcio Petenusso, Claudio Leone, Edison N Fujiki, Hugo Macedo Junior, Carlos B de Mello Monteiro, Isadora L Moreno, Ana Clara CR Gonçalves, Luiz Carlos de Abreu

**Affiliations:** 1 Laboratório de Escrita Científica, Departamento de Morfologia e Fisiologia; 2 Departamento de Cirurgia Ortopédica, Faculdade de Medicina do ABC. Av. Príncipe de Gales, 821. 09060-870, Santo André, SP, Brasil; 3 Departamento de Medicina, Disciplina de Cardiologia, Universidade Federal de São Paulo (UNIFESP). Rua Napoleão de Barros, 715 - Térreo. 04039-032, São Paulo, SP, Brasil; 4 Departamento de Fisioterapia, Faculdade de Ciências e Tecnologia, UNESP. Rua Roberto Simonsen, 305. 19060-900 Presidente Prudente, São Paulo, Brasil; 5 Departamento de Fonoaudiologia, Faculdade de Filosofia e Ciências, UNESP. Av. Higyno Muzzi Filho, 737. 17525-900 Marília, SP, Brasil; 6 Escola de Artes, Ciência e Humanidades da Universidade de São Paulo (USP). Av. Arlindo Béttio, 1000. 03828-000 São Paulo, SP, Brasil

## Abstract

**Background:**

The literature indicated that the majority of professional ballet dancers present static and active dynamic range of motion difference between left and right lower limbs, however, no previous study focused this difference in non-professional ballet dancers. In this study we aimed to evaluate active movements of the hip in non-professional classical dancers.

**Methods:**

We evaluated 10 non professional ballet dancers (16-23 years old). We measured the active range of motion and flexibility through Well Banks. We compared active range of motion between left and right sides (hip flexion and abduction) and performed correlation between active movements and flexibility.

**Results:**

There was a small difference between the right and left sides of the hip in relation to the movements of flexion and abduction, which suggest the dominant side of the subjects, however, there was no statistical significance. Bank of Wells test revealed statistical difference only between the 1^st ^and the 3^rd ^measurement. There was no correlation between the movements of the hip (abduction and flexion, right and left sides) with the three test measurements of the bank of Wells.

**Conclusion:**

There is no imbalance between the sides of the hip with respect to active abduction and flexion movements in non-professional ballet dancers.

## Background

Dance is a healthy activity which helps people to improve their life quality [1-3]. Dancers are a unique blend of artist and athlete particularly susceptible to musculoskeletal injuries and pain. The health problems of dancers are worthy of attention for several reasons. First, because most dancers begin training at a young age, there is potential for a great impact on their future health. Second, the interplay of physical and aesthetic demands in dance may lead to various health issues especially relevant to dancers. For example, a variety of musculoskeletal disorders have been described in athletes [4-7] and dancers [8-10] due to lower limbs overload, which may significantly impact on their health-related quality of life. In addition, biomechanical analysis evidenced range of motion difference between left and right lower limbs regarding force and flexibility. Moreover, previous studies showed impaired balance between right and left sides regarding passive and active movements in professional ballet dancers [11]. Finally, as an occupational group, non-professional dancers have received little attention in the health literature [10-12].

Dance is a "high-risk" activity with high incidence of musculoskeletal impairments. Yearly injury rates at ballet companies range from 67-95% [13]. Overuse injuries are related to the majority (60-76%) of all dance injuries [13]. Some dancers appear to be at higher risk of injury than others. At one classical ballet company, one group of dancers averaged 6.7 injuries each, versus an average of 1.86 injuries in the remaining injured dancers [14]. However, the authors did not investigate the ballet dancers training frequency.

A previous review [15] indicates that musculoskeletal injuries are relevant matter for dancers at all expertise stages. It was observed high prevalence and incidence of lower extremity and back injuries, with soft tissue and overuse injuries predominating. Several potential risk factors for injury were indicated by the literature [15]. On the other hand, conclusive evidence for any of these is lacking. Therefore, it led us to hypothesize that classical ballet experience in long term caused lower limbs range of motion difference also in non professional ballet dancers.

The literature suggests a relationship between hamstring flexibility and hip range of motion [16]. Previous investigations suggest that the concentric contraction of gluteus maximus is expected to control hip flexion or stabilize pelvis and prevent stance leg collapse by acting to extend the hip [17,18]. Thus, we believe that the investigation of this relationship is important to direct ballet training, since hamstring flexibility and hip range of motion are strictly related to dancers' performance [8-10].

As mentioned above, although it is indicated that the majority of professional ballet dancers present static and active dynamic range of motion difference between left and right lower limbs [19-21], no previous study focused this difference in non-professional ballet dancers. Therefore, this study aimed to evaluate the active movements of the hip in non-professional classical dancers. We used goniometry to measure range of motion and we applied the Bank of Wells test in order to analysis flexibility.

## Methods

### Subjects

Considering the difference between male and female subjects with respect to muscle movement and mass, we selected only female non-professional ballet dancers (n = 10) between 16 and 23 years old, non-smokers without cardiorespiratory compromise, non-sedentary and dancing ballet twice or three times a week and at least with 7 years of practice (average 7.6 years, ranging between 7 and 8 years). We included subjects that considered ballet as a recreational activity undertaken for relaxation or pleasure, typically done during one's leisure time, not as a primary activity. The study protocol was approved by the ethics committees of our University (number 004/08) and written informed consent was obtained from all voluntaries prior to enrolment.

### Range of motion (ROM) measurement

**Figure 1:**
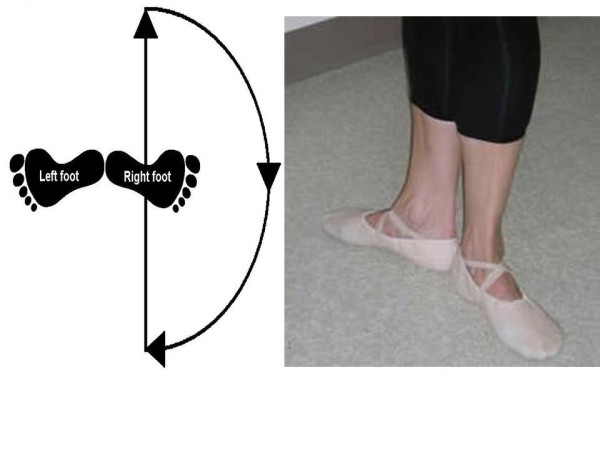
**"En dehor" position**.

One researcher measured the active range of motion (ROM) of classical non professional dancers at the supine position; we studied active hip abduction and flexion. The right side was the first side to be measured, each movement was measured three times; we calculated the mean of the three measurements. The movement of the hip abduction was carried out in rotation side, because most of the ballet exercises are held at that position, known as "en dehor", a position which requires the maximum lateral rotation of the hip, which further complicates and specifies ballet training [22] (Figure 1). The ROM of hip flexion and abduction was carried out with the knee extended. The differences between Steinberg et al method [20] and our method is that in their study they measured active hip abduction in a natural position and not combined with lateral rotation and the measurement of hip flexion was performed in flexed knee. We evaluated hip abduction and flexion because hip abduction combined with lateral rotation is held at "en dehor" position and hip flexion is a movement used by ballet dancers during many movements at standing.

### Analysis of flexibility

**Figure 2:**
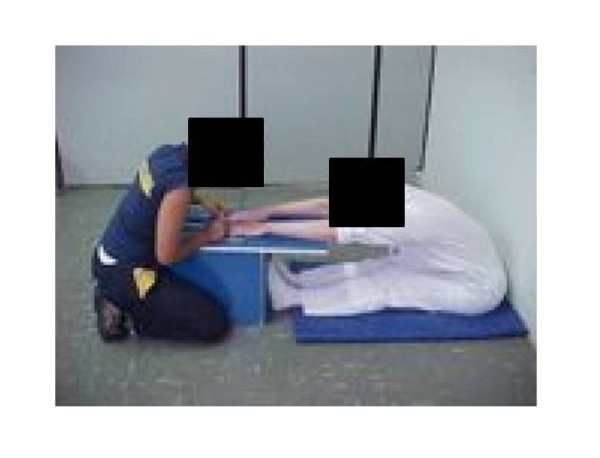
**Bank of Wells test**.

In order to analyze the flexibility of the volunteers, we applied the bank of Wells test, an indirect method to measure hip flexibility. The subject was positioned with the spine and hip on a wall or support, forming an angle of 90° with the legs, which were maintained extended. At that position, the subject extended her arms in front of the body, with the shoulders parallel to the ground and protruded hands outstretched fingers touching the tip of the piece of wood moves, without losing contact with the wall or with support. This position is the zero point in ruler for measuring the bank of Wells test. Thereafter, the subject pushed the piece of wood moving at the point closest to the platform in three attempts, trying to reach the highest distance point in the box of wood (Figure 2). We considered the highest value. The length was graded in cm based on the point that the volunteer reached and the joint evaluated was the hip [23,24].

### Statistical analysis

We applied the Kolmorogov Smirnov normality test for analysis of the distribution of the study population. In order to compare left and right sides of the hip in relation to flexion and abduction we applied the paired Student t test. For comparison of 3 values of the bank of Wells test, we applied one way ANOVA followed by Newmans-Keuls posttest. In order to verify the correlation between active ROM and bank of Wells performance, we applied the Pearson correlation coefficient; the highest value of ROM was correlated to each value of the bank of Wells. We also performed the Pearson correlation test between years practicing and the highest value of each subject in the Bank of Wells. The significance was considered only for p < 0.05.

## Results

Table 1 presents data regarding the demographic profile of our study population.

**Table 1:** Anthropometric data of non-professional ballet dancers

*Variables*	*Values*
***Height (m)***	1.60 ± 0.1
***Weight (kg)***	54.1 ± 8
***Body mass index (BMI) (kg/m^2^)***	21.1 ± 3

**Figure 3:**
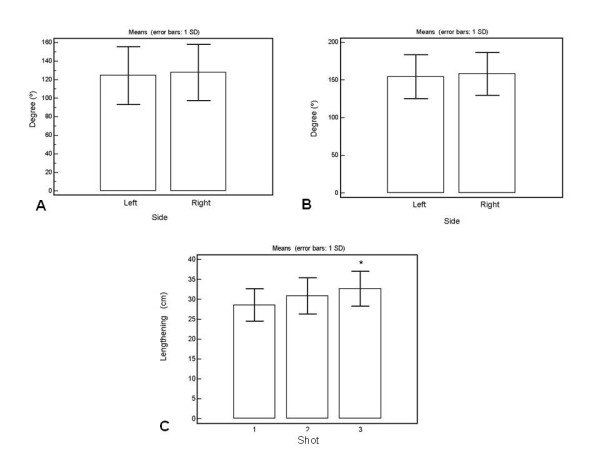
**Comparison between left and right side regarding abduction of the hip (A) and hip flexion (B)**. It also presented the performance of the ballet dancers in the Bank of Wells test (C). *p < 0.05: The 3^rd ^measurement is different from the 1^st^.

As shown in Figure 3A (comparison between right and left sides with regard to abduction of the hip: 122 ± 18° vs. 121 ± 18°; p > 0.05, respectively) and Figure 3B (comparison between the right and left sides with respect to hip flexion: 152 ± 42° vs. 151 ± 43°; p > 0.05, respectively), there was a small difference between the right and left sides of the hip in relation to the movements of flexion and abduction, however, there was no statistical significance. Figure 3C presents bank of Wells test performance, which revealed statistical difference only between the 1^st ^and the 3^rd ^measurement (p < 0.05).

We may observe in Table 2 the correlation between the movements of the hip (abduction and flexion, right and left sides) with the three test measurements of the bank of Wells. There was no significant association between the two measurements.

**Table 2:** Correlation between hip movements and Bank of Wells test performance

*Movement*	*R*	*p value*
***Abduction R***	-0.026	0.943
***Abduction L***	-0.037	0.99
***Flexion R***	0.54	0.10
***Flexion L***	0.54	0.10

R: right hip; L: left hip.

In order to verify if the long-term ballet practice may lead to overuse injuries or range of motion deficits we performed a correlation test between years practicing and the highest value of each subject in the bank of Wells. We observed no significant correlation between those variables (r = -0.2857; p = 0.64).

## Discussion

Our investigation aimed to evaluate active hip abduction and flexion in female non-professional ballet dancers. The group was composed by ballet dancers with at least 7 years of experience. We expected that due to the load that non-professional ballet dancers are exposed, even though it is lower compared to professional ballet dancers, we would find significant differences between the right and left sides regarding hip flexion and abduction. Nonetheless, when we compared the two sides of the hip as the movement of flexion and abduction we noticed a slight difference between the sides, which suggested the dominant side of each dancer. Nevertheless, these results did not reach statistical significance. With respect to flexibility, we used the bank of Wells test and we observed that the third measurement was significantly higher than the first. We believe that it was observed because after warming up and stretch-out of muscle/ligament it gradually increases. On the other hand, dancers work very hard to improve their flexibility, so we wonder if it compensates any potential adaptations to long-term dance practice.

Our data suggest that non-professional ballet dancers are exposed to lower load compared to professional ballet dancers. According to Hincapié et al [15], there was evidence that musculoskeletal impairment is an important health issue for dancers at all skill levels. There was high prevalence and incidence of lower extremity and back injuries, with soft tissue and overuse injuries predominating. Several potential risk factors for injury were suggested by the literature, but conclusive evidence for any of these is lacking. Although we did not perform a deeper evaluation, in our study population, we may highlight the main following risk factors for musculoskeletal disorders: constant loads the subjects are exposed, flexibility and stress caused by load exposure. Taken together, it led us to speculate that ballet experience in long term caused lower limbs range of motion difference also in non professional ballet dancers. However, our research did not report such difference, since there was no significant difference between right and left active hip flexion and abduction. We believe that the reduced load that the non professional dancers are exposed (compared to professional dancers) may explain the absence of range of motion difference regarding active movements.

In order to verify if the posterior muscle chain (femoral biceps, semimembranosus and semitendinosus muscles) was associated with active hip abduction and flexion, we performed a correlation between left and right hip abduction and flexion with the three measurements of the bank of Wells. There was no significant association between hip movements and the bank of Wells test performance. Nevertheless, it was previously suggested that the concentric contraction of gluteus maximus, which is prolonged during important ballet movements at standing, is expected to control hip flexion or stabilize pelvis and prevent stance leg collapse by acting to extend the hip [17,18]. Therefore, we were expecting significant correlation between the two variables because we hypothesized that higher active flexibility of posterior muscle chain (measured by the bank of Wells test) would be associated with higher active range of motion regarding hip flexion and abduction. Perhaps, a better investigation of this muscle complex could help us to better understand the absence of correlation between bank of Wells performance and hip movements.

We measured the correlation between range of motion of the hips and hamstring and low-back flexibility (bank of Wells test) in order to verify if hip abduction and hip flexion are associated with indirect flexibility tested in the bank of Wells test. We believe that a positive association between range of motion and hamstring and low-back flexibility would provide important information in order to direct ballet training. Nonetheless, our findings suggest that flexibility measured by the indirect bank of Wells test is not associated with active hip abduction and flexion.

In our study we examined non-professional ballet as a recreational activity that is undertaken for pleasure or relaxation, typically done during one's leisure time; hence, this population is of great interest. If we consider the implications of ballet practice for overuse injuries, we should also consider how much a professional dancer practices compared to a non-professional dancer. While professional ballet dancers practice at least five days per week, eight hours per day, non-professional ballet dancers practice around two-three days per week, up to three hours per day. Thus, professional ballet dancers are exposed to lower limbs overload in a higher intensity.

Our report presents interesting findings, although non professional ballet dancers are exposed to lower limbs overload and previous investigations indicated that the majority of professional ballet dancers present range of motion difference between left and right lower limb passive and active movements [15,19,20], suggesting a possible difference between left and right passive and active movements in non professional dancers, we indicated that there was no significant difference between left and right side regarding hip active movements in non professional ballet dancers.

## Conclusion

There is no imbalance between the sides of the hip with respect to the active movements of abduction and flexion in non professional ballet dancers. Future studies may also consider the effect of gender and age and influence of other muscles that act on the hip movements in non professional ballet dancers.

## Competing interests

The authors declare that they have no competing interests.

## Authors' contributions

EEV, VEV and LCA participated in the acquisition of data and revision of the manuscript. EEV, VEV, CF, OFMF, LCMV, TDC, NT, MP, CL, ENF, HMJ, CBMM, ILM, ACCR and LCA conceived of the study, determined the design, performed the statistical analysis, interpreted the data and drafted the manuscript. All authors read and gave final approval for the version submitted for publication.
